# Blood donation practice and predictors among university and college students in Ethiopia: A systematic review and meta-analysis

**DOI:** 10.1016/j.puhip.2025.100687

**Published:** 2025-12-11

**Authors:** Hailemariam Gezie, Mekuriaw Wuhib, Fekadeselassie Belege Getaneh, Habtam Gelaye

**Affiliations:** a Department of Emergency and Critical Care Nursing, College of Health Sciences, Debre Tabor University, Debre Tabor, Ethiopia; b Department of Comprehensive Nursing, College of Medicine and Health Sciences, Wollo University, Dessie, Ethiopia; c Department of Pediatrics and Child Health Nursing, College of Medicine and Health Sciences, Wollo University, Dessie, Ethiopia; d Department of Psychiatry, College of Medicine and Health Sciences, Wollo University, Dessie, Ethiopia

**Keywords:** Blood donation practice, University and college students, Systematic review, Meta-analysis

## Abstract

**Objectives:**

This systematic review and meta-analysis aimed to assess the pooled blood donation practice and its predictors among university and college students in Ethiopia.

**Study design:**

Systematic review and meta-analysis.

**Methods:**

Multiple databases and search engines, such as PubMed, African Journals Online, Hinari, Google Scholar, and repositories, were searched using search terms created by combining Medical Subject Heading words and phrases for each database. A total of 1306 articles were found, and after removing duplicates and other irrelevant articles, 22 articles were included. Relevant data were extracted using a standardized Excel template and analyzed using STATA 17 software. The prevalence of blood donation practice and its predictors were pooled using a random effects model. Statistical heterogeneity was identified using the Galbraith plot, I^2^, and Q statistic and handled by subgroup analysis, meta-regression, and sensitivity analysis. Publication bias was checked by funnel plot and Egger's test.

**Results:**

This systematic review and meta-analysis of 22 studies that included 9048 students revealed that the pooled estimate of blood donation practice was 26 % (CI: 22, 31). Age of students (POR = 3.22; CI: 1.83, 5.68), faculty (POR = 2.44; CI: 1.74, 3.41), knowledge (POR = 2.89; CI: 1.89, 4.41), and attitude (POR = 1.93; CI: 1.43, 2.62) were found to have a significant association with blood donation practice.

**Conclusion:**

The pooled estimate of blood donation practice indicated that only a quarter of university and college students donated blood, which is limited. Therefore, Ethiopian Ministry of Health, regional health bureaus, blood banks, the universities and colleges, the students’ council, and other stakeholders shall pay due attention to blood donation.

## Highlights

• Blood donation practice of Ethiopian students is inadequate.
• The pooled estimate shows only a quarter of students donated blood (26 %, CI: 22, 31).
• Age, health faculty, Knowledge and attitude toward blood donation were positive predictors of blood donation.

## 1 Introduction

Blood is usually an essential resource for the healthcare system to achieve better patient outcomes, and people can save millions of lives worldwide by donating safe blood [1,2]. Blood donation is an act of altruism and kindness in which a healthy individual gives blood for transfusion purposes [[3], [4], [5]]. About 118 million blood donations are collected worldwide annually, most of which are from high-income countries, up to nine times more than low-income countries [[6], [7], [8]]. Most of these donations are utilized for maternal and childbirth conditions, severe childhood illnesses such as thalassemia and sickle cell disease, patients with anemia, and other emergency conditions like trauma and surgery [1,2,[6], [7], [8]] Although the World Health Organization recommends that at least 1 % of the population has to donate blood to meet a country's blood demand and the highest contributors should be adults, the young adult population accounts for the least representation [9]. Many low- and middle-income countries are suffering from shortages of blood [10,11], and the demand for blood transfusions is high in Africa due to different conditions such as bleeding from pregnancy and childbirth, severe anemia, an increased rate of motor vehicle accidents, and other injuries and surgical procedures [12,13]. Therefore, increasing the number of volunteer donors is very important to ensure an adequate and safe blood supply. In this regard, university students represent an accessible population with the potential for long-term donation commitment and are considered ideal candidates for regular voluntary blood donation [14].

Students are the best and most easily accessible sources of blood donation [5], and they are ideal, energetic, healthy, and have long-term potential prospects for donation [10,15]. However, the rate of blood donation from voluntary donors is still low among young adults [16,17], particularly students of higher education institutions, despite the increased need for blood transfusions in Ethiopia for different health issues.

Blood donation practice among university and college students varies across different countries. In East Africa, studies done in Sudan [18], Kenya [19], and Somalia [20] revealed that 11 %, 54.5 %, and 65 % of university and college students donated blood, respectively. A study done in Egypt also revealed only 35 % of 576 students had ever donated blood [15]. Additionally, among 840 students in Nigeria, only 7.3 % of them donated blood [21]. The highest prevalence of blood donation practice in Africa was revealed by a study conducted in Nigeria, which revealed 71.3 % of 300 students had ever donated blood [22]. A multicounty study done in 16 countries in Africa, Asia, and Europe involving 12,606 university students revealed that only 22.7 % of them had donated blood at least once [10]. A study conducted in China revealed that only 24.71 % of 5168 university students donated blood [23]. Studies conducted among 398 North American and Caribbean students [24] and 364 female students in the United states of America [25] revealed that 63.2 % and 49 % of students had ever donated blood, respectively.

Understanding the level of blood donation practice among university and college students is vital for increasing the rate of donations and voluntary non-remunerated donors among students. However, no systematic review and meta-analysis show particularly the pooled blood donation practice and predictors among students in Ethiopia, except a meta-analysis that explored the pooled estimate of blood donation practice among the general population [26]. In this systematic review and meta-analysis, we explored the pooled blood donation practice and its predictors among university and college students. Therefore, this study aimed to fill the gap by incorporating insights from the previous research findings and providing targeted recommendations to enhance blood donation efforts.

## 2 Methods

### 2.1 Data source and search strategy

This systematic review and meta-analysis adhered to the Preferred Reporting Items for Systematic Reviews and Meta-Analysis (PRISMA-2020) standards [27] (Supplementary Material 1). To ensure wider coverage of relevant studies, multiple databases and search engines, such as PubMed, African Journals Online, Hinari, Google Scholar, and repositories, were searched extensively. Additionally, a snowball search of references of included studies was performed. MeSH (Medical Subject Heading) words and phrases were combined to create a search strategy for each database. The following search terms were used: (“Blood donation"[Title/Abstract]) OR (“Voluntary blood donation"[Title/Abstract]) OR (“Blood donation practice"[Title/Abstract]) AND (Predictors[Title/Abstract]) OR (Determinants[Title/Abstract]) OR (“Associated factors"[Title/Abstract]) AND (“University and College Students"[Title/Abstract]) OR (“University students"[Title/Abstract]) OR (“College students"[Title/Abstract]) OR (Students[Title/Abstract]) AND (Ethiopia [Title/Abstract]).

### 2.2 Study design

Systematic review and meta-analysis.

### 2.3 Inclusion and exclusion criteria

All observational studies published in English before December 31, 2024, that reported the blood donation practice and its predictors among university and college students in Ethiopia were included in this systematic review and meta-analysis. Those studies focused on particular demographic characteristics, case reports, case series, letters to the editor, studies that did not report the prevalence of blood donation practice, and meta-analyses were excluded.

### 2.4 Outcome of review

The outcome variable of the review is the blood donation practice and associated factors among university and college students in Ethiopia.

### 2.5 Study selection

All the studies searched using various databases and search engines were exported into Endnote X9. Duplicate articles were removed, and then the title, abstract, and full text of each article were examined independently by two authors (HG and Habtam G). Disagreements between the two authors were resolved through discussion with the third author (MW), who then screened all of the articles for eligibility.

### 2.6 Data extraction and management

Data were extracted from each included study using a standardized data extraction template prepared in Microsoft Excel. Information such as the last name of each author, year of publication, type of institutions where the primary studies were conducted (government, private, or both), field of study (faculty), design of the study, sampling technique, sample size, the proportion of blood donation practice, and associated factors in odds ratio (OR) with their confidence interval was extracted by the two authors (HG and FBG) independently. Disagreements were resolved by discussion with the other author (Habam G).

### 2.7 Quality assessment

The Joanna Briggs Institute (JBI) quality appraisal tool, which was modified for observational studies, was used to assess the quality of the included studies [28]. The tool has eight items. The first item was about criteria for inclusion in the sample, the second was about study subjects and the setting, the third was about exposure measurement, the fourth was about measurement of the condition, the fifth was about confounding factors, the sixth was about strategies to deal with confounding factors, the seventh was about outcomes measurement, and the eighth was about statistical analysis used with responses Yes, No, Unclear, or Not Applicable. It was assessed by three reviewers (Habtam G, FBG, and MW) independently. Any discrepancy between the three reviewers’ results was resolved by discussion with the third reviewer (HG).

### 2.8 Data synthesis and analysis

After extracting the data using Microsoft Excel format, it was exported to STATA version 17 software for analysis. The pooled blood donation practice among university and college students in Ethiopia was estimated using a random effects model [29]. Statistical heterogeneity between the included studies was identified using the Galbraith plot, forest plot, I^2^, and Q statistics [30]. Subgroup analysis was performed by publication period, field of study, study design, type of institution, sampling method, sample size, and study quality to reduce the variance of point estimates between studies. Meta-regression was also done. Additionally, sensitivity analysis was done to control the effect of a specific study on the pooled estimate. To identify publication bias, a funnel plot and Egger's test were executed [31]. The pooled odds ratio with a 95 % confidence interval was computed to determine the factors associated with blood donation practice among university and college students in Ethiopia.

**Systematic review registration:** The review has been registered in PROSPERO with an ID number of CRD42024586763. No amendment was made to the protocol after registration.

## 3 Results

### 3.1 Identification and characteristics of included studies

**Fig. 1:**
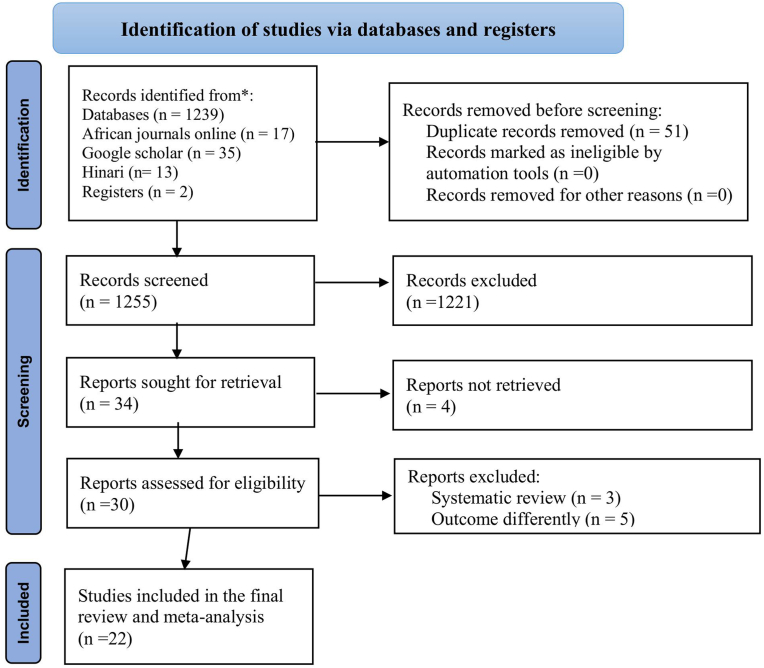
PRISMA 2020 flow diagram for a systematic review and meta-analysis of blood donation practice among university and college students in Ethiopia.

One thousand three hundred six (1306) primary studies were found in the initial search of both published articles and gray literature until October 12, 2024. Among these articles, 1239 were from PubMed, 17 were from African journals online, 35 were from Google Scholar, 13 were from Hinari, and two were from another source. These documents were imported into the EndNote X9 citation manager. Of the total articles searched, 51 were found to be duplicates and removed. The other 1221 articles were excluded by their titles and abstracts, and 34 articles were retrieved for full text. Finally, 22 studies that included 9048 university and college students fulfilled the inclusion criteria and were included in the final meta-analysis (Fig. 1).

Of the 22 articles, nine studies [[32], [33], [34], [35], [36], [37], [38], [39], [40]] were conducted among health science students, ten studies [12,[41], [42], [43], [44], [45], [46], [47], [48], [49]] were conducted among both health and non-health science students, and the other three studies [[50], [51], [52]] were conducted among non-health science students. 17 studies [[32], [33], [34],[36], [37], [38], [39], [40],[42], [43], [44],[46], [47], [48], [49], [50], [51]] were conducted in governmental higher education institutions, and three studies [12,41,52] were done in private institutions. Regarding sampling techniques, participants of seven studies [34,36,37,43,46,48,51] and eight studies [33,35,39,40,42,45,49,52] were selected using stratified and simple random sampling techniques, respectively (Table 1).

**Table 1:** Characteristics of the studies included in this systematic review and meta-analysis.

Author	Pub/year	Field of study	Institution type	Study design	Sampling technique	Sample size	Prevalence	Quality score
Misganaw C et al. [32]	2014	HSC	Govt	CS	Systematic	384	0.234	8
Nigatu A et al. [42]	2014	Both^a^	Govt	CS	Simple R	399	0.236	5
Ayene BA et al. [43]	2020	Both^a^	Govt	CS	stratified	334	0.254	8
Talie E et al. [44]	2020	Both^a^	Govt	CS	Multistage	619	0.168	8
G/selassie H et al. [33]	2017	HSC	Govt	CS	Simple R	360	0.25	4
Melku M et al. [34]	2018	HSC	Govt	CS	stratified	255	0.125	6
Idris E et al. [45]	2023	Both^a^	Both^b^	CS	Simple R	518	0.357	6
Kebede Z et al. [41]	2021	Both	Private	CS	Multistage	349	0.344	4
Shamebo T [50]	2020	Non-HSC	Govt	CS	Systematic	346	0.147	6
Aschale A et al. [12]	2021	Both^a^	Private	CS	Multistage	595	0.491	6
Darega B et al. [46]	2015	Both^a^	Govt	CS	stratified	609	0.184	6
Dejen M et al. [35]	2021	HSC	Both^b^	CS	Simple R	412	0.124	7
Tadesse W et al. [36]	2018	HSC	Govt	CS	stratified	339	0.245	6
Yosef T et al. [37]	2020	HSC	Govt	CS	stratified	394	0.355	8
Shama A et al. [47]	2022	Both^a^	Govt	CS	Systematic	360	0.408	8
Obsa MS et al. [38]	2018	HSC	Govt	CS	Consecutive	96	0.167	6
Teklu T et al. [51]	2020	Non-HSC	Govt	CS	stratified	363	0.231	8
Mussema A et al. [48]	2024	Both^a^	Govt	CS	stratified	393	0.193	6
Tenaw A et al. [52]	2021	Non-HSC	private	CC	Simple R	392	0.33	8
Baye Z et al. [49]	2019	Both^a^	Govt	CS	Simple R	814	0.256	5
Gebre BG et al. [40]	2024	HSC	Govt	CS	Simple R	315	0.432	7
Regassa DA et al. [39]	2024	HSC	Govt	CS	Simple R	407	0.287	6

**HSC** = Health science; **both^a^** = Health science and non-health science; **Govt** = government; **Both^b^** = both government and Private; **CS** = Cross-Sectional; **CC** = Case-Control; **Simple R** = simple random sampling.

### 3.2 Quality of included studies

The total score of included studies in the quality assessment varied from 4 to 8. Studies with a score of <4 were of low quality, studies with a score of 4–5 were of moderate quality, and studies with a score of 6–8 were of high quality. Of the 22 studies, eighteen studies [12,32,[34], [35], [36], [37], [38], [39], [40],[43], [44], [45], [46], [47], [48],[50], [51], [52]] had high quality, and four studies [33,41,42,49] had moderate quality. No included study was found to have low quality (Supplementary material 2) (Table 1).

### 3.3 Pooled estimate of blood donation practice among students in Ethiopia

**Fig. 2:**
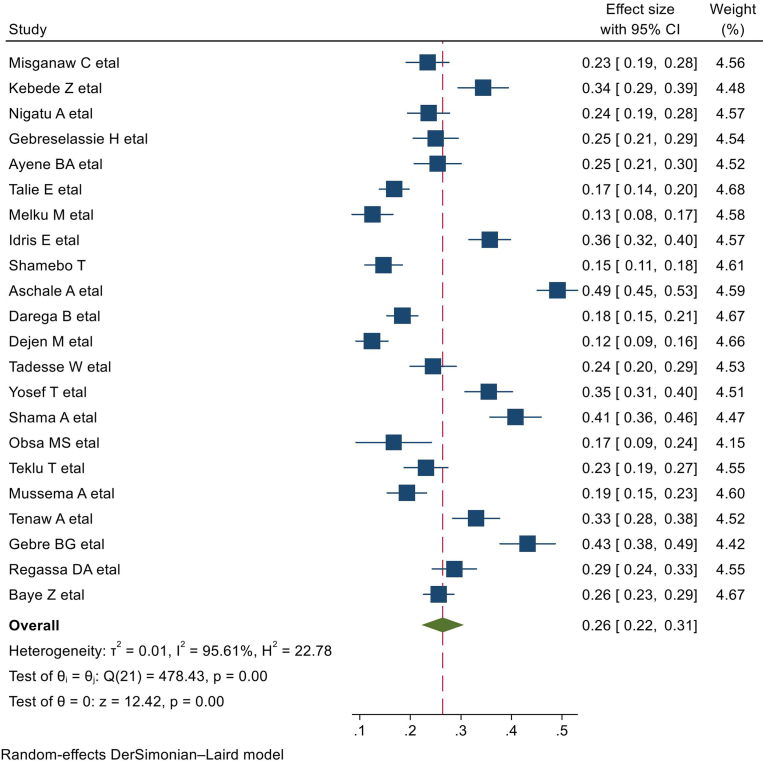
Forest plot for the pooled blood donation practice among university and college students in Ethiopia.

This systematic review and meta-analysis revealed that the pooled estimate of blood donation practice among students was 26 % (CI: 22, 31) in the 22 studies conducted among students in Ethiopian higher education institutions. However, there was high heterogeneity from variations between the included studies (I^2^ = 95.61 %, p = 0.00) (Fig. 2).

### 3.4 Handling heterogeneity

Heterogeneity was checked by the Galbraith plot, forest plot, and I^2^ and Q statistics. To handle the heterogeneity, subgroup analysis, meta-regression, and sensitivity analysis were done in the random effect model.

### 3.5 Galbraith test

The Galbraith plot was done, and it indicates one study was outside the confidence interval, which indicated the presence of heterogeneity (Supplementary material 3).

### 3.6 Subgroup analysis

A subgroup analysis was done for the publication period, field of study, type of institutions where primary studies were done, study design, sampling method, sample size, and quality of studies. A subgroup analysis by publication period revealed that the highest pooled blood donation practice among university and college students was seen in studies done since 2020 and later (29 %; CI: 23, 36). The highest pooled estimate of blood donation practice was seen among private students, which was 39 % (CI: 28, 50). Additionally, the highest pooled estimate was revealed in studies that utilized multistage sampling techniques (33 %; CI: 13, 54) compared to others. The pooled estimate of blood donation practice by moderate-quality studies (27 %, CI: 23, 31) was slightly higher than the pooled estimate of those studies with high quality (26 %, CI: 21, 31) (Table 2).

**Table 2:** Sub-group analysis by Publication period, field of study, owner of institution, Study design, sampling method, sample size, and quality of studies.

Subgroup		Number of studies	Pooled prevalence (%) with 95 % CI	Heterogeneity		
				I^2^	Q(DF)	P-value
Publication period	Before 2020	8	21(18, 255)	81.21 %	7(37.25)	0.00
	2020 and after	14	29(23, 36)	96.83 %	13(410.10)	0.00
Field of study	HSC	8	25 (17, 32)	95.54 %	7(156.96)	0.00
	Non-HSC	3	24(13, 34)	94.80 %	2(36.37)	0.00
	Both^a^	11	28(22, 34)	96.23 %	10(265.04)	0.00
Type of institutions	Governmental	17	24(21, 28)	92.55 %	16(214.64)	0.00
	Private	3	39(28, 50)	93.98 %	2(33.25)	0.00
	Both^b^	2	24(1, 47)	98.70 %	1(76.81)	0.00
Study design	Cross sectional	21	26(0.22, 30)	95.71 %	20(465.85)	0.00
	Case control	1	33(28, 38)	–	–	–
Sampling method	Systematic	3	26(12, 41)	96.97 %	2(65.95)	0.00
	Multistage	3	33(13, 54)	98.80 %	2(166.55)	0.00
	Simple R	8	28(22, 35)	95.04 %	7(141.03)	0.00
	Stratified	7	23(18, 28)	90.41 %	6(62.51)	0.00
	Consecutive	1	17(9, 24)	–	–	–
Sample size	≥400	15	26(22, 31)	93.14 %	14(204.06)	0.00
	<400	7	27(18, 35)	97.80 %	6(273.02)	0.00
Quality of studies	High	18	26(21, 31)	96.33 %	17(462.66)	0.00
	Moderate	4	27(23, 31)	75.57 %	3(12.28)	0.0

**HSC** = Health science; **other** = non-health science; **both^a^** = Health science and non-health science; **Govt** = government; **Both^b^** = both government and Private; **Simple R** = simple random sampling.

### 3.7 Meta-regression

Meta-regression analysis in the random effect model was also done on study variables to examine heterogeneity further and indicated that publication period (R-squared = 0.84 %; p = 0.067), Field of study (R-squared = 0.00 %; p = 0.346), study design (R-squared = 0.00 %; p = 0.502), sampling method (R-squared = 0.00 %; p = 0.451), sample size (R-squared = 0.00 %; p = 0.053), and quality of studies (R-squared = 0.00 %; p = 0.878) were not found to be significantly associated with blood donation practice and had no contribution to the heterogeneity. However, the type of institution had a significant association with blood donation practice (R-squared = 17.74 %; p = 0.037). This indicates that 17.74 % of the heterogeneity between studies was explained by the type of institutions [53] (Table 3).

**Table 3:** Meta-regression analysis for the pooled estimate of blood donation practice among university and college students in Ethiopia.

Variables	Coefficient	Standard error	R-squared (%)	P > [z]	95 % CI
Publication period	0.0806	0.0440	0.84	0.067	−0.0057, 0.1670
Field of study	−0.0284	0.0301	0.00	0.346	−0.0875, 0.0307
Type of institutions	0.0851	0.0407	**17.74**	**0.037**	−0.0053, 0.1650
Study design	−0.0691	0.1030	0.00	0.502	−0.2709, 0.1326
Sampling method	−0.0140	0.0186	0.00	0.451	−0.0504, 0.0224
Sample size	0.0027	0.0468	0.00	0.953	−0.0889, 0.0944
Quality of study	0.0087	0.0703	0.00	0.878	−0.1020, 0.1195

### 3.8 Sensitivity analysis

**Fig. 3:**
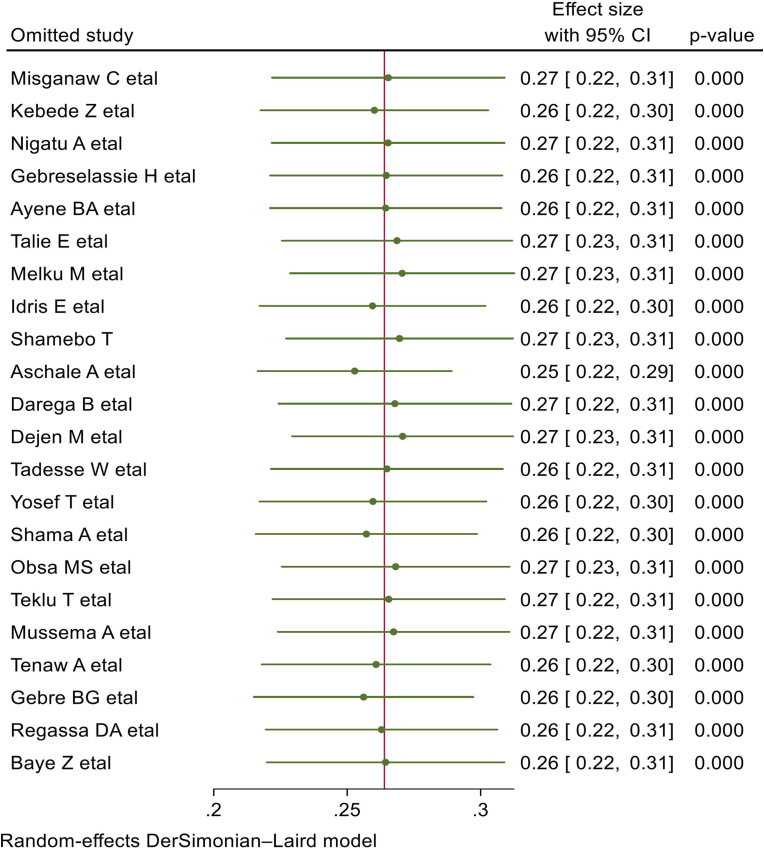
Sensitivity analysis of the pooled estimate of blood donation practice among university and college students in Ethiopia.

A leave-one-out sensitivity analysis was done to identify the source of heterogeneity in the pooled estimate of blood donation practice. However, there was no point-estimated prevalence outside the confidence interval of the pooled estimate of blood donation practice when each study was left out from the analysis. This shows that the pooled estimate of blood donation practice among students could be trustworthy. The pooled prevalence in the sensitivity analysis ranges from 25 % to 27 % (Fig. 3).

### 3.9 Publication bias

**Fig. 4:**
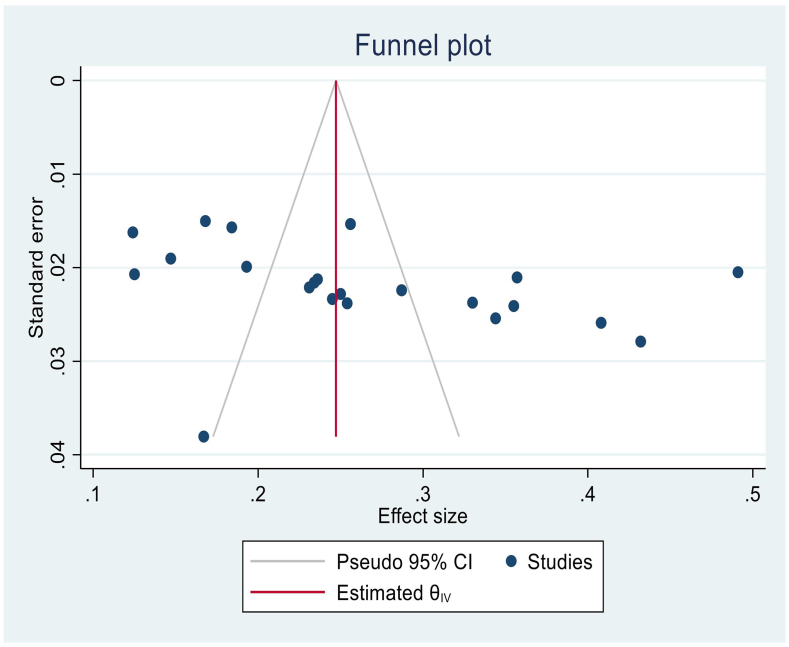
Funnel pot test to check publication bias for t the pooled estimate of blood donation practice among university and college students in Ethiopia.

To check for a publication bias, a standard funnel plot and Egger's tests were performed. The funnel plot seems to show an asymmetrical distribution of the scatter plots (Fig. 4). Therefore, an Egger's test was also done, and publication bias was not evident (p = 0. 1344)**.**

### 3.10 Factors associated with blood donation practice

Those variables that had a significant association with blood donation practice in three or more primary studies were included in this meta-analysis. These variables, such as gender, age, faculty, knowledge, and attitude, were eligible.

The meta-analysis of three studies [33,44,45] revealed no association between sex and blood donation practice among university and college students (POR = 0.74; CI: 0.28, 1.945; I^2^ = 88.75 %; P = 0.00). The pooled result of three studies [32,34,35] in this meta-analysis indicated that students who were in the age group of 25 years and above had a significant association with donation practice among university and college students in Ethiopia (POR = 3.22; CI: 1.83, 5.68; I^2^ = 11.59 %; P = 0.32) compared to those who were in the age group of 18–24 years. Additionally, the pooled result of four studies [36,41,45,48] in this meta-analysis indicated that health science students had a significant association with donation practice (POR = 2.44; CI: 1.74, 3.41; I^2^ = 0.00 %; P = 0.76) compared to those students in other disciplines. Similarly, the pooled result of eight studies [35,37,41,42,44,45,47,48] in this meta-analysis showed that university and college students who had good knowledge of blood donation had increased blood donation practice (POR = 2.89; CI: 1.89, 4.41; I^2^ = 82.82 %; P = 0.00) compared to those students who had poor knowledge. However, there was heterogeneity among the studies. Moreover, the pooled result of five [35,37,46,47,52] in the random effect model showed a strong association between favorable attitude and blood donation practice among university and college students (POR = 1.93; CI: 1.43, 2.62; I^2^ = 0.00; P = 0.94) (Supplementary material 4).

## 4 Discussion

According to this systematic review and meta-analysis, the pooled estimate of blood donation practice among Ethiopian university and college students from 22 studies was 26 % (CI: 22, 31). This indicates that the involvement of students in blood donation is limited, posing additional challenges to ensuring a sustainable blood supply to meet national transfusion demands. As a result, regular blood donation campaigns and other activities that encourage students to participate in blood donation practices are quite important.

This pooled estimate was consistent with a systematic review and meta-analysis conducted in Ethiopia, which found 25.82 % of Ethiopians had ever donated blood [26]. This similarity could be attributed to Ethiopians’ shared social and cultural features. The healthcare systems, healthcare access and coverage, and the research design were also similar in Ethiopia among different age groups and demographic characteristics.

However, this finding was slightly higher than a subgroup analysis of six studies that included 2009 students from a previous meta-analysis done in Ethiopia [26], which reported the pooled blood donation practice of 20.63 % among students of higher education institutions. This difference might be due to the difference in the number of primary studies included in the two meta-analyses, in which the current study included 22 studies and the previous study included only six studies. The other reason might be the number of students included in the two meta-analyses, in which the current study included 9048 students and the previous one included only 2009 students. Additionally, the period between the two studies is different, in which there have been many technological, healthcare advocacy, and access to information advancements over the past five years in Ethiopia. In recent years, students have had a better chance to learn and know more about blood donation from many digital sources, such as social media, artificial intelligence, and web browsers. It was also higher than a multi-country study done in 16 countries found in Africa, Asia, and Europe [10], which revealed 22.7 % of 12,606 university students had donated blood at least once, and studies conducted in Sudan (11 %) [18], Nigeria (7.3 %) [21], (15.3 %) [54], Gaza (16.75 %) [55], Saudi Arabia (14.6 %) [56], (23.6 %) [57], Syria (14.0 %) [58], Jordan (11.3 %) [59], India (12 %) [60], (18 %) [61], and China (24.71 %) [23]. This discrepancy might be because of differences in study design, sample size, sampling method, sociodemographic conditions, knowledge, and attitude levels among students. The healthcare and blood bank systems might also differ among the above countries.

On the other hand, our finding was lower than the findings revealed by studies conducted in Kenya 54.5 % [19], Somalia 65 % [62], Eritrea 34.35 % [63], Uganda 39.4 % [64], Tanzania 45.5 % [23], Egypt 35 % [15], Nigeria 71.3 % [22], Saudi Arabia 29.0 % [65], North America and the Caribbean 63.2 % [24], and the USA 49 % [25]. This inconsistency might be due to differences in students' social, cultural, demographic, and economic characteristics. The number of study participants, sampling technique, study design, and study settings are also different. Additionally, the students’ opportunity to learn about blood donation and related issues in their curricula may also be quite different.

The pooled result of three studies in this meta-analysis indicated that students who were in the age group of 25 years and above were three times more likely to donate blood compared to those who were in the age group of 18–24 years. This finding was supported by findings from a multi-country study conducted in 16 countries [10] and a study conducted in southern Tanzania [66]. This may be because older students may have increased awareness, experience, and exposure to blood donation campaigns, which further increase their knowledge, attitude, and willingness to donate blood compared to younger students [5,67,68]. Older age may also have better altruism, kindness, and social responsibility [69].

The pooled result of four studies indicated that health science students were 2.44 times more likely to donate compared to those students in other disciplines. This was supported by studies done in Iraq [70], Nepal [71], and Italy [72]. This can be explained by the health science students may be more knowledgeable and aware than other students about the importance of blood donation. They may be more informed about the medical implications of blood donation and transfusion requirements in healthcare facilities compared to students in different disciplines.

Additionally, the pooled result of eight studies in our meta-analysis showed that university and college students who had good knowledge of blood donation had 2.89 times increased blood donation practice compared to those students who had poor knowledge. This finding was supported by studies conducted in Nigeria [73,74], Nepal [75], Bangladesh [76], Pakistan [77], India [78], South Korea [79], and a multi-country study done in 16 countries [10]. Students with good knowledge can have good awareness, avoid misconceptions about blood donation, foster a positive attitude, and may have the ability to withstand barriers that can be obstacles to donating blood.

Moreover, the pooled result of five studies in the random effects model showed that there were 1.93 times increased blood donation practices among students who had a favorable attitude compared to those with an unfavorable attitude. It was supported by studies conducted in Tanzania [80], Nigeria [22,73], Pakistan [77], and South Korea [79]. The possible explanation could be that students with a positive attitude may engage in blood donation, as the positive attitude fosters willingness to donate blood, motivation, and altruism. An altruistic motivation can influence the students’ decision-making on blood donation. A positive attitude is also linked with social responsibility and encouraging students to act on their beliefs and donate blood.

### 4.1 Strengths and limitations

This systematic review and meta-analysis was the first study in Ethiopia that reported the blood donation practice of higher education institution students, which included all published and unpublished data. Rigorous efforts were taken to overcome heterogeneity among included studies using subgroup analysis, meta-regression, and sensitivity analysis. The possible limitations of this review could be variability among the included studies and a relatively small number of studies were included, which could affect its generalizability.

### 4.2 Conclusions

This systematic review and meta-analysis indicated that the pooled estimate of blood donation practice among university and college students was too low despite students being potential donors. The review also revealed that the age of students, faculty, knowledge about blood donation, and attitude towards blood donation were significant predictors of blood donation practice. Therefore, the Ethiopian Ministry of Health, regional health bureaus, blood banks, the universities' and colleges' administration, students' council, and other stakeholders shall pay due attention to blood donation among students through different activities such as awareness creation and blood collection campaigns in the university and college compounds. The universities and colleges should also create, support, and strengthen blood donation clubs to increase the students’ engagement in regular blood donation campaigns.

Author statements

## Ethics statement

This systematic review and meta-analysis summarizes data from the existing research findings, which had no direct relation with individual participants, and the results from this meta-analysis did not use personal identifiers. Therefore, ethics approval is not applicable for this review work.

## Authors’ contributions

HG and Habtam G designed the review idea. HG outlined the search strategies. All the authors participated in the literature search and quality appraisal. HG, Habtam G and MW extracted the data from included studies. HG and FBG performed the analysis and interpretation. HG wrote the manuscript draft, and all authors reviewed the final version of the manuscript and approved it.

## Availability of data and materials

All data regarding systematic review and meta-analysis are incorporated in the manuscript.

## Funding

No funding was obtained.

## Declaration of competing interest

We the authors declare that we have no known competing financial or other interests and personal relationships that could influence the work reported in this manuscript.
