# Supplementary material for: Blood donation practice and predictors among university and college students in Ethiopia: A systematic review and meta-analysis
Source: Public Health Pract (Oxf). 2025 Dec 11;11:100687. doi: 10.1016/j.puhip.2025.100687 (PMC12771492; doi:10.1016/j.puhip.2025.100687)
Supplement: Multimedia component 1 [file mmc1.docx]

Summary of quality assessments using JBI quality appraisal checklist

| **Author and publication year** | **Q1** | **Q2** | **Q3** | **Q4** | **Q5** | **Q6** | **Q7** | **Q8** | **Score** |
| --- | --- | --- | --- | --- | --- | --- | --- | --- | --- |
| Misganaw C etal[32] | 1 | 1 | 1 | 1 | 1 | 1 | 1 | 1 | 8 |
| Kebede Z etal [41] | 0 | 1 | 1 | 0 | 1 | 0 | 0 | 1 | 4 |
| Nigatu A etal[42] | 0 | 1 | 1 | 1 | 0 | 0 | 1 | 1 | 5 |
| G/selassie H etal[33] | 1 | 1 | 0 | 0 | U | U | 1 | 1 | 4 |
| Ayene BA etal[43] | 1 | 1 | 1 | 1 | 1 | 1 | 1 | 1 | 8 |
| Talie E etal [44] | 1 | 1 | 1 | 1 | 1 | 1 | 1 | 1 | 8 |
| Melku M etal[34] | 1 | 1 | 1 | 1 | 0 | 0 | 1 | 1 | 6 |
| Idris E etal[45] | 1 | 1 | 1 | 1 | 0 | 0 | 1 | 1 | 6 |
| Shamebo T[50] | 1 | 1 | 1 | 1 | 0 | 0 | 1 | 1 | 6 |
| Aschale A etal[12] | 1 | 1 | 1 | 1 | 0 | 0 | 1 | 1 | 6 |
| Darega B etal[46] | U | 1 | 1 | U | 1 | 1 | 1 | 1 | 6 |
| Dejen M etal [35] | 1 | 1 | 1 | U | 1 | 1 | 1 | 1 | 7 |
| Tadesse W etal[36] | U | 1 | 1 | 1 | 1 | 0 | 1 | 1 | 6 |
| Yosef T etal[37] | 1 | 1 | 1 | 1 | 1 | 1 | 1 | 1 | 8 |
| Shama A etal[47] | 1 | 1 | 1 | 1 | 1 | 1 | 1 | 1 | 8 |
| Obsa MS etal[38] | 1 | 1 | 1 | 1 | N | N | 1 | 1 | 6 |
| Teklu T etal[51] | 1 | 1 | 1 | 1 | 1 | 1 | 1 | 1 | 8 |
| Mussema A etal[48] | 1 | 1 | 1 | 1 | 0 | 0 | 1 | 1 | 6 |
| Tenaw A etal[52] | 1 | 1 | 1 | 1 | 1 | 1 | 1 | 1 | 8 |
| Baye Z etal [49] | 0 | 1 | 1 | 0 | 1 | 0 | 1 | 1 | 5 |
| Gebre BG etal [40] | 1 | 1 | 1 | 0 | 1 | 1 | 1 | 1 | 7 |
| Regassa DA etal [39] | 1 | 1 | 1 | 1 | 0 | 0 | 1 | 1 | 6 |

JBI Criteria to be scored:

- Q1. Were the criteria for inclusion in the sample clearly defined?
- Q2. Were the study subjects and the setting described in detail?
- Q3. Was the exposure measured in a valid and reliable way?
- Q4. Were objective, standard criteria used for measurement of the condition?
- Q5. Were confounding factors identified?
- Q6. Were strategies to deal with confounding factors stated?
- Q7. Were the outcomes measured in a valid and reliable way?
- Q8. Was appropriate statistical analysis used?

**Abbreviations:** 1 = Yes; 0 = No; U = Unclear; NA = Not Applicable; JBI: Joanna Briggs Institute

**Criteria used to rank the risk of bias**

- <50% = high risk of Bias
- 51% - 70% = Moderate risk of Bias (4 studies)
- Above 70% = low risk of Bias (17 studies)
