# Supplementary material for: Blood donation practice and predictors among university and college students in Ethiopia: A systematic review and meta-analysis
Source: Public Health Pract (Oxf). 2025 Dec 11;11:100687. doi: 10.1016/j.puhip.2025.100687 (PMC12771492; doi:10.1016/j.puhip.2025.100687)
Supplement: Multimedia component 2 [file mmc2.docx]

**Figure:** Galbraith plot for pooled estimate of blood donation practice among university and college students in Ethiopia
