# Supplementary material for: Blood donation practice and predictors among university and college students in Ethiopia: A systematic review and meta-analysis
Source: Public Health Pract (Oxf). 2025 Dec 11;11:100687. doi: 10.1016/j.puhip.2025.100687 (PMC12771492; doi:10.1016/j.puhip.2025.100687)
Supplement: Multimedia component 3 [file mmc3.docx]

**Association between gender, age, faculty, and attitude with blood donation practice**

1. **Association between gender and blood donation practice**

**Figure 6:** Association between sex and blood donation practice among university and college students in Ethiopia

1. **Association between age and blood donation practice**

**Figure 7:** Association between age and blood donation practice among university and college students in Ethiopia

1. **Association between Faculty and blood donation practice**

**Figure 8:** Association between age and blood donation practice among university and college students in Ethiopia

1. **Association between Knowledge and blood donation practice**

**Figure 9:** Association between knowledge and blood donation practice among university and college students in Ethiopia

1. **Association between attitude and blood donation practice**

**Figure 10:** Association between social support level and occurrence of depression among prisoners in Ethiopia
